# A randomized, controlled field study to assess the efficacy and safety of lotilaner flavored chewable tablets (Credelio™) in eliminating fleas in client-owned dogs in the USA

**DOI:** 10.1186/s13071-017-2469-x

**Published:** 2017-11-01

**Authors:** Daniela Karadzovska, Kimberly Chappell, Shane Coble, Martin Murphy, Daniela Cavalleri, Scott Wiseman, Jason Drake, Steve Nanchen

**Affiliations:** 1 Elanco Animal Health, Yarrandoo, NSW Australia; 2 Elanco Animal Health, 2500 Innovation Way, Greenfield, IN 46140 USA; 3 Elanco Animal Health, Mattenstrasse 24a, 4058 Basel, Switzerland; 4 Elanco Animal Health, Basingstoke, Hants UK

**Keywords:** Credelio, Lotilaner, Fleas, Afoxolaner, Dogs, Field study

## Abstract

**Background:**

Preclinical studies have shown that the novel isoxazoline, lotilaner (Credelio™, Elanco) administered orally to dogs, produces rapid flea and tick knockdown and sustained speed of kill for at least a month post-treatment with a wide safety margin. A field study was undertaken to validate pre-clinical results.

**Methods:**

Dogs were enrolled at 10 veterinary clinics across the United States. Qualifying households containing up to three dogs and one primary dog with at least 10 fleas were randomized 2:1 to receive lotilaner (Credelio™, Elanco) at the recommended minimum dose of 20 mg/kg, or afoxolaner (Nexgard®, Merial), administered per label, to give a minimum dose of 2.5 mg/kg. Treatments were dispensed on Days 0, 30 and 60 for administration by owners; all household dogs received the same treatment as the primary dog. Post-enrollment flea and tick counts were made on primary dogs on Days 30, 60 and 90, and all dogs were assessed for tablet palatability and safety.

**Results:**

For efficacy assessments, data were used from 111 lotilaner-treated dogs and 50 afoxolaner-treated dogs; for safety, 197 and 86 dogs, respectively. Percent reductions from baseline in geometric mean flea counts for the lotilaner group were 99.3, 99.9 and 100% on Days 30, 60 and 90, respectively, and for afoxolaner 98.3, 99.8 and 99.8% (*P* < 0.001, both groups, all days). On Day 90, 100% of lotilaner-treated dogs and 93% of afoxolaner-treated dogs were flea-free. Too few ticks were present to allow assessment. There were no differences in palatability between products (*P* = 0.2132), with, respectively, 94% and 96% of lotilaner and afoxolaner treatments accepted when offered by hand, in an empty food bowl or with food. Both treatments were well tolerated, alleviating clinical signs of flea allergy dermatitis (FAD) in dogs affected at enrollment.

**Conclusion:**

A single owner-administered lotilaner treatment was greater than 99% effective in reducing mean flea counts within 30 days. Three consecutive monthly lotilaner treatments resulted in a 100% reduction in flea infestations, and a substantial reduction in signs of FAD. Lotilaner flavored tablets were readily accepted under field conditions. The absence of treatment-related adverse events confirms the safety of lotilaner in dogs.

**Electronic supplementary material:**

The online version of this article (10.1186/s13071-017-2469-x) contains supplementary material, which is available to authorized users.

## Background

Pruritus is a common outcome of flea infestations in dogs, and can progress to alopecia and more severe dermatological conditions, including flea allergy dermatitis (FAD) [1]. In some dogs, the injection of flea saliva as the parasite feeds can lead to an overall increase in antigenic load and result in atopic flares in susceptible dogs [2]. Fleas have been shown to be vectors of zoonotic diseases, such as those caused by *Rickettsia* spp. and *Bartonella* spp., and are intermediate hosts of the tapeworm *Dipylidium caninum* which can develop into adult stages in children who ingest infected fleas [3, 4]. In uncontrolled flea infestations, contamination of household premises can also lead to flea bite problems in exposed humans [4].

The female flea is a prolific egg layer, and under laboratory conditions has been shown to begin egg laying within 24 to 36 h of finding a host, and then to lay as many as 50 eggs per day, with daily egg production continuing over a life-time of more than 100 days [5]. Flea eggs falling from a host animal then provide potential for an enormous increase in immature life-cycle stages in the environment. In the absence of effective treatments these stages continue to develop to present an increasing flea challenge. Use of environmental pesticides to eliminate flea populations from a household may not be effective, and also risks exposure of household inhabitants to the pesticide [6]. It is therefore important that infested animals be treated with products that eliminate existing flea burdens and provide protection against post-treatment challenges from a contaminated environment.

The novel isoxazoline, lotilaner, provides veterinarians and their clients with a fast-acting and lasting effective measure to control canine flea and tick infestations. In safety and efficacy studies lotilaner was shown to be well tolerated, including in a study in which doses of up to 215 mg/kg, 1 day per month (each daily dose more than 10 times the minimum recommended dose) over 3 months were administered to puppies that were 8 weeks old at the initial treatment [7–11]. In laboratory studies, lotilaner was shown to begin killing fleas and ticks within 4 h after treatment [8, 9]. Efficacy against fleas and ticks was then sustained through 35 days after treatment, indicating that monthly use of lotilaner will be effective in causing the depletion of flea life-cycle stages from a dog’s environment [10, 11].

A field study was designed to confirm the results of the pre-clinical development studies. The primary objective of the study was to evaluate the efficacy and safety of lotilaner flavored chewable tablets administered orally by dog owners at a targeted minimum dose rate of 20 mg/kg for the treatment and control of flea infestations. Secondary objectives were to assess the presence and persistence of the clinical signs associated with FAD (pruritus, erythema, scaling, papules, alopecia, and dermatitis/pyodermatitis), to evaluate the acceptance of the formulation, and in the event of a tick infestation, to evaluate activity against ticks on naturally infested dogs.

## Methods

This was a randomized, double-blind, positive controlled field study with dogs enrolled at veterinary practices across the United States. The protocol was prepared in compliance with the World Association for the Advancement of Veterinary Parasitology (WAAVP) guidelines for evaluating the efficacy of parasiticides for the treatment, prevention and control of flea and tick infestation on dogs and cats [12]. The study was conducted and documented in accordance with US Code of Federal Regulations, Title 21, Part 511, Section 511.1, *New Animal Drugs for Investigational Use Exempt from Section 512(a) of the Act* (April 2013) and the United States Food and Drug Administration - Center for Veterinary Medicine (FDA/CVM) Guidance for Industry 85, International Co-operation on Harmonisation of Technical Requirements for Registration of Veterinary Products (VICH) GL9, *Good Clinical Practice* (May 2001).

### Animals and households

For enrollment into the study, a household was required to contain at least one and no more than three dogs, all of which had to be at least 8 weeks of age, weigh at least two kilograms, and be clinically healthy or have minor ailments judged not to interfere with the study. Dogs with chronic diseases (i.e. diabetes, hypothyroidism, osteoarthritis) considered to be stable or controlled were eligible for inclusion. At least one dog in the household was required to have at least 10 fleas.

Households were excluded for the following reasons: if they contained dogs that were intended for breeding, or that were pregnant or lactating; if there had been any environmental flea treatment in the 3 months prior to the study. A household would also be excluded if it contained dogs on concurrent treatment likely to interfere with the conduct or interpretation of study results (e.g. treatment with another ectoparasiticide preparation). The minimum time of withdrawal for such treatments corresponded to the efficacy duration given on the label. If the product applied was not clearly identified, the minimum time of withdrawal was 4 weeks. If the treatment applied was a collar, the minimum time of withdrawal was 2 weeks prior to study participation. To avoid any potentially confounding factors that might have impacted on the Day 0 flea counts, there was an exclusion for bathing/shampooing study dogs within 48 h prior to treatment.

Other than for that pretreatment restriction, or use of any product active against fleas and/or ticks, there were no restrictions on wetting or bathing and no restrictions on the presence of non-canine household pets. There was no stipulation on whether dogs were kept indoors or outdoors. Cats in any study household were treated once monthly for the duration of the study with a commercially available monthly flea adulticide that was dispensed by the clinic at the time of dispensing study treatments.

The experimental unit was the primary dog in each household. If more than one dog in a household met all inclusion criteria, including a burden of at least 10 fleas, the first dog in terms of alphabetical order of each qualifying dog’s name was selected as the primary household dog. All dogs in the household receiving treatment and returning for at least one follow-up visit were included in the safety analyses. The dogs were client-owned and therefore were fed, housed and managed by their owners. Standard veterinary procedures were followed at each clinic. The owner was required to maintain study dogs on the same diet throughout the study.

Dogs could be withdrawn from the study at the discretion of the investigator, if an owner withdrew consent, or for any adverse event that required stopping study treatment or observations. Other reasons for withdrawal included administration of any protocol-forbidden concomitant treatment, lack of efficacy of either product, loss of the household to follow-up, and deviations from the protocol that might have compromised the integrity of the study.

### Enrollment

At each clinic, study dogs were weighed, and received a thorough physical examination including body condition scoring and, for any dog with at least 10 fleas, assessments of the signs of FAD. Blood was collected from all study dogs for hematology and blood chemistry testing, and urine was collected for urinalysis, as a baseline assessment of general health. All dogs were combed for flea and tick counts. When all dogs in a household were found to be eligible, with at least one dog with 10 or more fleas, the household was enrolled into the study. Within each clinic, primary dogs were then blocked into threes on order of household enrollment and randomly allocated to a treatment group in a 2:1 ratio of lotilaner to afoxolaner. Other household dogs, to a maximum of two supplementary dogs per household, were to receive the same treatments according to the same schedule as primary household dogs. Supplementary dogs were not assessed at subsequent visits for flea and tick counts, but were assessed for FAD if their flea count at the enrollment visit was at least 10, and if clinical signs of FAD were present at the enrollment visit. No further FAD assessments were made after Day 0 if there were no signs of FAD at the enrollment visit. The study targeted enrollment of 100 lotilaner primary dogs and 50 afoxolaner primary dogs.

### Treatments

Treatments dispensed to owners for administration at home were:

(i) Lotilaner (Credelio™, Elanco, Greenfield, IN, USA) available to each clinic for dispensing in four tablet sizes: 56.25 mg, 112.5 mg, 225 mg and 450 mg, to be administered on the basis of each household dog’s body weight at the recommended minimum dose rate of 20 mg/kg.
(ii) Afoxolaner (Nexgard®, Merial, Duluth, GA, USA) available to each clinic for dispensing in four tablet sizes: 11.3 mg, 28.3 mg, 68 mg and 136 mg, to be administered administered per label, to give a minimum dose of 2.5 mg/kg.

In each clinic, the examining veterinarian conducting the general physical examination, and assessing FAD and body condition score was blinded to treatments. The person(s) dispensing study treatments to owners was responsible for treatment group allocation, training the owner on treatment of the animals, and drug accountability. No treatment-related information was disclosed to the examining veterinarian (and/or trained designees), and the records were maintained separately from the examining veterinarian’s records. Blinding labels were placed on individualized blisters such that the label obscured any existing text on the blister so that the owners would remain blinded to treatment. At the initial visit and at the second and third visits, the dispenser in each clinic provided the appropriate number of tablets for each household dog to be treated once on each of Days 0, 30 (± 2), and 60 (± 2). Owners were instructed to feed their dogs within approximately 30 min prior to treatment.

Each dog’s owner was instructed to initially offer the tablet by hand for approximately 90 s. If the dog did not accept and consume the tablet from the hand, the tablet was to be placed in the dog’s empty bowl for approximately 90 s. If the dog still did not eat the tablet, it was to be offered with a small amount of food for approximately 90 s. If this was not successful, the owner was to administer the tablet directly into the dog’s mouth, on the back of the tongue, and then to encourage the dog to swallow. If the tablet was vomited within 60 min of administration, the owner was instructed to contact the investigator so that a replacement could be provided.

Concomitant treatments were allowed as long as they did not interfere with the objectives of the study. Some concomitant medications, such as corticosteroids, antihistamines, and antibiotics given for FAD signs, required exclusion of the FAD assessment data. Routinely administered/dispensed products such as vaccinations, heartworm preventives, intestinal parasiticides or nutritional supplements were acceptable.

### Flea and tick counting and assessment of flea allergy dermatitis

Flea comb counts were performed on Days 0, 30, 60 and 90 for the primary dog in each household. At each visit, the number of ticks found during combing was also recorded. Fleas and ticks were counted manually by combing the entire body of each dog for approximately 20 min using a fine-toothed flea comb. At the initial visit, if fewer than 10 fleas were counted within the initial 5 min of combing, the counting was stopped. Infestations of greater than 250 fleas were recorded as > 250, and for such heavily infested dogs, the value of 251 was used for analysis. Fleas that were combed out were disposed of and not returned to any dog.

The examining veterinarian also assessed each study dog with a flea count of at least 10, on Day 0 and (± 2 days of) Days 30, 60 and 90 for signs of FAD, classifying each sign (pruritus, papules, erythema, alopecia, scaling, dermatitis/pyodermatitis) as Absent, Mild, Moderate, or Severe. Where possible the same veterinarian completed each follow-up assessment for each primary and supplementary dog in which any sign of FAD was present at the enrollment visit. For dogs with no clinical signs associated with FAD on Day 0, no additional FAD assessments were conducted on Days 30, 60 and 90, but the subsequent appearance of such signs was recorded as an adverse event.

### Assessments and statistics

The efficacy of each treatment in the control of flea infestation was assessed by comparing baseline flea counts on Day 0 with those at 30, 60 and 90 days after the enrollment visit. Efficacy was determined on the basis of the percent reduction in live adult flea counts from pre- to post-dosing within each treatment group. Percent efficacy at each counting time point after dosing was calculated as follows:

$$ \mathrm{Percent}\  \mathrm{efficacy}=\left(\left[\mathrm{MB}-\mathrm{MA}\right]/\mathrm{MB}\right)\times 100, $$

where MB is the mean flea count prior to dosing (Day 0) and MA is the mean flea count post-dosing (Day 30, 60 and 90).

Calculations were completed using geometric means for efficacy determination, and arithmetic means were also calculated. Calculation of geometric means involved taking the logarithm of the flea count of each dog. If any of the flea counts were equal to zero, a one was added to the count for every animal in the group and then subtracted from the resultant mean prior to calculating percent efficacy.

For each treatment group, the log-transformed flea (count +1) data were analyzed from pre- to post-dosing (SAS procedure PROC MIXED) to determine if a statistically significant flea count reduction from baseline occurred at each time point. The model included a fixed effect *Paired* which was defined as an indicator variable (0, 1; to represent pre- and post-dosing counts), and also included the random effects of Site. Separate models were fitted for each of Days 30, 60 and 90 compared to Day 0. Either treatment was considered to be effective at each time point if the following criteria were met: (i) The animals were adequately infested with fleas prior to dosing (≥ 10 fleas); (ii) The calculated efficacy at the time point was ≥ 90%; (iii) There was a statistically significant decrease at a 2-sided 0.05 level of significance (*P* < 0.05) in pre- to post-dosing flea counts at the time point.

This study was designed to meet regulatory requirements, and between-group comparisons were not a study objective. However, treatment group comparisons were completed at each time point with respect to flea count and the proportion of dogs free of fleas. Separate models were fitted for each of Days 30, 60 and 90. To compare the flea counts, the log transformed flea (count +1) data were analyzed (SAS procedure PROC MIXED) with treatment group and baseline flea count as fixed effects and site as a random effect. The proportion of dogs with zero fleas was compared between the two treatment groups using Fisher’s exact test.

A total FAD score was calculated for each animal at each time point as the sum of the clinical sign scores (pruritus, erythema, scaling, papules, alopecia, dermatitis/pyodermatitis scores). and was evaluated over time using SAS procedure PROC MIXED. Differences within a treatment group over time were determined through the LSMEANS statement.

For assessment of the relative palatability of each study product, a repeated measures generalized linear mixed model was fitted. The model utilized a binomial response distribution and logit link function. Tablet palatability (accept/not accept) was the response variable. Treatment group, study day (Day 0, 30, 60) and treatment group by study day interaction were fixed effects. Since each dog was dosed multiple times the correlation between successive observations on the same dog was incorporated into the model. The treatment group main effect was used to test the null hypothesis of no difference in the acceptance rate of the tablets across the entire duration of the study between the treatment groups. Confidence intervals for the acceptance rate in each group were also calculated.

### Translations

Spanish translation of the article is available in Additional file 1. French translation of the Abstract is available in Additional file 2.

## Results

From July to December, 2014, 122 primary dogs were enrolled in the lotilaner group (214 in total including supplementary household dogs) and 58 primary dogs in the afoxolaner group (98 dogs in total) at 10 small animal veterinary clinics throughout the United States (one clinic in each of California, Florida, Georgia, Louisiana, Michigan, Missouri, Oregon, Pennsylvania, South Carolina and Texas). For the safety population, defined as enrolled dogs which received at least one study treatment and returned for at least one post-treatment visit, there were 197 and 86 dogs in the lotilaner and afoxolaner groups, respectively. The mean age of dogs in the safety population of both groups was approximately 5.5 years (Table 1). There was a similar distribution of age groupings, and approximately 82% of dogs in each group were older than 12 months. The minimum ages were 2 months in the lotilaner group and 3 months in the afoxolaner group, and minimum weights were 2.0 kg and 2.2 kg, respectively. The sex and neuter status of enrolled dogs was similar in both groups, as was the distribution of single and multiple dog households. Purebred dogs comprised 53.8% of dogs enrolled into the lotilaner group (36 different breeds) and 61.6% of dogs in the afoxolaner group (22 different breeds), with Chihuahuas and Labrador Retrievers the most frequently enrolled breeds in each group.

**Table 1:** Demographics of enrolled dogs and distribution of numbers of dogs in each household. Numbers based on safety population of study dogs, defined as enrolled dogs which received at least one study treatment and returned for at least one post-treatment visit

		Lotilaner (*n* = 197)	Afoxolaner (*n* = 86)
Age (years)	Mean ± SD	5.6 ± 4.0	5.5 ± 3.6
	Range	0.2–16.0	0.3–15.0
Weight (kg)	Mean ± SD	16.2 ± 11.4	18.8 ± 13.6
	Range	2.0–46.1	2.2–65.0
Sex	Female, intact	25 (12.7%)	13 (15.1%)
	Female, spayed	70 (35.5%)	32 (37.2%)
	Male, intact	48 (24.4%)	15 (17.4%)
	Male, neutered	54 (27.4%)	26 (30.2%)
Distribution of household sizes (number of dogs)	1	54 (46.6%)	25 (48.1%)
	2	43 (37.1%)	20 (38.5%)
	3	19 (16.4%)	7 (13.5%)

*Abbreviation*: *SD* Standard deviation

Of the 312 primary and supplementary dogs that were enrolled, 259 completed the study (179 in lotilaner-group households and 80 in afoxolaner-group households). Of the 312, 53 dogs (35 lotilaner; 18 afoxolaner) were prematurely terminated. For assessments of efficacy, data were used from 111 lotilaner-treated dogs and 50 afoxolaner-treated dogs. The analysis sets and cases excluded were defined in collaboration with FDA-CVM. Reasons for exclusion included loss of follow-up, occurrence of an adverse event that required stopping the observations, withdrawal of owner’s consent, and death (one primary dog and one secondary dog died, discussed below).

**Fig. 1:**
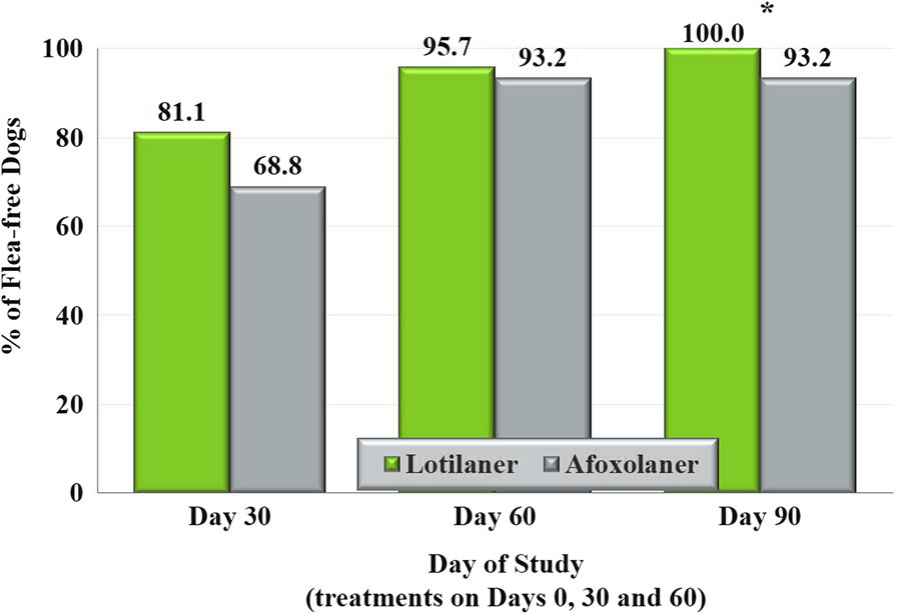
Percentage of primary dogs in each group that were flea-free at each post-treatment assessment on Days 36, 60 and 90. *Difference between groups significant, *P* = 0.0323

Both groups showed statistically significant (*P* < 0.001; Table 2) reductions in mean flea counts from baseline (pre-treatment, Day 0 visit) to the end of the study. At this point 100% of lotilaner-treated dogs and 93% of afoxolaner-treated dogs were free of fleas (Fig. 1). This difference in proportions of dogs free of fleas between the two treatment groups was statistically significant (*P* = 0.0323, table probability = 0.0323) (Table 3). The statistical comparison of the treatment groups also showed that there were significantly fewer fleas on dogs in the lotilaner group than in the afoxolaner group on Days 30 (*t*_(143)_ = 2.63, *P* = 0.0095) and 90 (*t*_(125)_ = 2.37, *P* = 0.0193). Percent reductions in geometric mean flea counts for the lotilaner group were at least 99% on Days 30, 60 and 90, and for the afoxolaner group were greater than 99% only on Days 60 and 90 (Table 2).

**Table 2:** Flea count data for each treatment group and statistical analysis of flea count reduction from baseline for each treatment group

		Lotilaner	Afoxolaner
Day 0	Arithmetic mean ± SD	70.7 ± 75.0	62.3 ± 61.8
	Range^a^	10–251	10–251
	Geometric mean	42.3	41.8
Day 30	Arithmetic mean ± SD	0.7 ± 2.5	2.3 ± 5.5
	Range	0–20	0–25
	Geometric mean	0.3	0.7
	% reduction	99.3	98.3
	Statistical analysis	*t*_(105)_ = 35.50, *P* < 0.001	*t*_(47)_ = 21.25, *P* < 0.001
Day 60	Arithmetic mean ± SD	0.1 ± 0.4	0.3 ± 1.8
	Range	0–3	0–12
	Geometric mean	0.0	0.1
	% reduction	99.9	99.8
	Statistical analysis	*t*_(93)_ = 38.19, *P* < 0.001	*t*_(43)_ = 28.10, *P* < 0.001
Day 90	Arithmetic mean ± SD	0.0 ± 0.0	0.1 ± 0.6
	Range	0–0	0–4
	Geometric mean	0.0	0.1
	% reduction	100	99.8
	Statistical analysis	*t*_(173)_ = 37.07, *P* < 0.001	*t*_(43)_ = 27.49, *P* < 0.001

*Abbreviation*: *SD* standard deviation

^a^Any count > 250 was assigned the value 251

**Table 3:** Statistical analysis comparing treatment groups with respect to geometric mean flea counts and proportion of dogs with zero fleas

	Flea count		Dogs with zero fleas	
Day 30	*t*_(143)_ = 2.63	*P* = 0.0095	Table prob. = 0.0396	*P* = 0.1001
Day 60	*t*_(127)_ = 1.44	*P* = 0.1530	Table prob. = 0.2493	*P* = 0.6795
Day 90	*t*_(125)_ = 2.37	*P* = 0.0193	Table prob. = 0.0323	*P* = 0.0323

Too few dogs presented with ticks to make any study assessment of efficacy valid. Therefore, there was no analysis of tick counts.

Both study products were well accepted when administered by dog owners. For lotilaner tablets, 94% were accepted when offered by hand, in an empty food bowl or with food. Only 6% of dogs in the lotilaner group and 4% in the afoxolaner group received the tablet directly into the mouth, and 100% of treatments were successfully administered for both groups. There were no significant effects for Treatment group (*F*_(1,281)_ = 1.56, *P* = 0.2132), Study day (*F*_(2,524)_ = 1.41, *P* = 0.2458) or the interaction between group and study day (*F*_(2,524)_ = 0.01, *P* = 0.9885). No dogs were reported to have vomited within 1 h of treatment, and no dogs required redosing with either product.

Between Days 0 and 30, 26 (12.1%) lotilaner-group dogs and 19 (19.4%) afoxolaner-group dogs received treatment for FAD and were therefore ineligible for ongoing FAD assessments. There were 55 dogs in the lotilaner group and 29 in the afoxolaner group in which clinical signs of FAD (pruritus, papules, erythema, alopecia, scaling, dermatitis/pyodermatitis) were present at baseline and were then followed throughout the study. At baseline, pruritus and erythema were jointly the most prevalent signs of FAD observed in the lotilaner group (41 dogs with each sign, i.e. 33.6% of the primary dogs enrolled), with one dog recorded with severe pruritus. Improvement was seen in 40 of the 41 dogs during the course of the study and by Day 90 the most severe signs were recorded as mild only. Pruritus and erythema were also the most prominent signs in the afoxolaner group, affecting 24 (41.4%) and 21 (36.2%) dogs on Day 0, respectively. A significant reduction in the overall presence and persistence of FAD signs occurred in both treatment groups by Day 30 (lotilaner *t*_(129)_ = 9.79, *P* < 0.001; afoxolaner *t*_(131)_ = 6.67, *P* < 0.001), when 52 (94.5% of dogs showing signs at baseline) dogs in the lotilaner group and 25 (86.2%) in the afoxolaner group showed improvement. By Day 90, signs of FAD had resolved or improved and remained significantly lower than baseline on Days 60 (lotilaner *t*_(142)_ = 10.46, *P* < 0.001; afoxolaner *t*_(141)_ = 7.98, *P* < 0.001) and 90 (lotilaner *t*_(142)_ = 10.47, *P* < 0.001; afoxolaner *t*_(141)_ = 8.14, *P* < 0.001).

The percent of lotilaner-group dogs with at least one reported adverse event over the three study periods, Day 0–30, Day 30–60, and Day 60–90, was 10.7, 8.1 and 9.1%, respectively, compared to 16.3, 12.9 and 3.8% in dogs receiving afoxolaner. Across the 3 months of the study, adverse events were observed in 24.4% of lotilaner group dogs (13.6% of doses administered) and 26.7% of afoxolaner group dogs (16.5% of doses administered). The majority of these events were classified as Skin and Appendages Disorders (primarily linked to dermatitis) observed in 7.1% of lotilaner-treated dogs and 9.3% of afoxolaner-treated dogs. A number of dogs in each group showed isolated events of appetite changes and lethargy, plus some localized edema reported for dogs receiving afoxolaner. Observations of digestive tract disorders, including vomiting and diarrhea, occurred at a low rate in each group, involving 5.1% of dogs treated with lotilaner and 7.0% of those receiving afoxolaner. The incident rate of adverse events, including adverse events involving dermatitis, declined in both groups from Day 0 to the final assessment. There were significant differences in both groups between the baseline and Day 90 study exit indices for several hematology, clinical chemistry and urinalysis parameters. However, all arithmetic means remained within normal ranges, and while some values fell outside the clinical pathology reference ranges, these were not considered clinically relevant by the study investigators.

The primary dog that died was a seven-year-old Yorkshire terrier in the lotilaner group that had radiological signs of cardiomegaly and a diagnosis of chronic obstructive pulmonary disease at enrollment. This dog’s condition deteriorated and at post-euthanasia necropsy a malignant splenic histiocytoma was found. The secondary dog that died, also in the lotilaner group, was a 13-year-old Pomeranian that went into respiratory distress midway between the second and scheduled third visits. At enrollment, this dog had severe periodontal disease combined with 3+ proteinuria and elevated blood urea nitrogen/creatinine (BUN/Cr), suggestive of underlying renal disease. The owner reported the dog’s death 2 weeks after the report of respiratory distress and no follow-up was possible. Neither of the events in these two dogs was considered to be related to treatment.

In the afoxolaner group, one primary dog, a five-year-old border collie/Labrador cross, suffered two mild to moderate convulsions, each approximately 1 month after receiving the scheduled study treatment. This dog remained in the study. The adverse event that resulted in withdrawal of an afoxolaner-treated dog was an incident of lethargy, vomiting and diarrhea from which the dog recovered and which the investigator considered unrelated to treatment. Other serious adverse events that occurred in study dogs included renal and urinary disorders, abscess, and digestive tract disorders (one in each group). The relationship of these disorders to study treatments was considered by the respective investigators to be unknown or unlikely, and none of the withdrawals was attributed to treatment.

A range of concomitant medications was administered to lotilaner-treated dogs, including those disallowed by the protocol, ranging across butorphanol, ketamine hydrochloride, multiple classes of antibiotics, anticonvulsants, non-steroidal anti-inflammatory drugs, oclacitinib, corticosteroids, macrolide and benzimidazole anthelmintics, and otic products. Rabies, *Bordetella* and multivalent vaccines were administered to over 10% of lotilaner-treated dogs, and all concomitant treatments appeared to be well tolerated.

## Discussion

The diverse genetic background of dogs enrolled in the lotilaner group (36 breeds plus mixed breed dogs), and their broad geographic spread across the different regions of the United States provide a solid representation of the real world in which an antiparasitic product would be used. Moreover, the summer to early spring timing of the study means that study households and dogs would have been exposed to seasonal factors which can precipitate dermatological conditions, and can increase exposure to flea infestations [2, 13]. Regardless of the field conditions, the results align with the laboratory studies that indicated lotilaner to be a safe and effective flea control treatment for dogs [7, 8, 10]. Both lotilaner and afoxolaner were shown to be highly effective from the first treatment onward. At every post-Day 0 assessment, mean flea counts, the percentage of infested dogs and the maximum flea count in any individual dog were consistently numerically lower in the lotilaner group than in the afoxolaner group. At the end of the study no fleas were found on any lotilaner-treated dog.

The scoring of FAD lesions as mild, moderate and severe, as used in this study, is consistent with that described for other field studies that assessed the effectiveness of introductory flea control products [14–16]. A limitation of this scoring methodology is that it has not been validated and it is subjective, and so grading may vary between clinicians. Nonetheless, as with other reports, the progressive and marked improvement in each clinical sign of FAD in treated dogs can be attributed to two factors related to lotilaner’s rapid onset and sustained residual speed of kill [8, 10]. One factor is the reduced antigenic challenge that results from the rapid knockdown of newly emerged fleas. Linked to that rapid knockdown, the second factor stems from the sustained speed of flea kill, which eliminates newly-emerged fleas from a dog before egg-laying can begin, thus allowing a progressive depletion of household flea biomass, leading to its complete elimination.

The results in this study align with those reported from a European field study, conducted according to a similar protocol, in which lotilaner efficacy in 128 primary dogs was 99.5, 99.9 and 99.8% on Days 28, 56 and 84, respectively, with 98.4% of treated dogs flea-free on Day 84 [17]. A similar protocol has been used to investigate the efficacy of other monthly, orally-administered flea control products. A study in the USA of another isoxazoline compound, sarolaner, compared the results of 195 sarolaner- and 98 spinosad-treated primary dogs. Efficacy of both products was of > 99% on Days 60 and 90 [18]. A European study also compared the performance of sarolaner with spinosad, enrolling 93 and 44 dogs in each group, respectively. Reported efficacy was 99.4, > 99.9 and > 99.9% in the sarolaner-treated group and 93.7, 96.8 and 95.1% in the spinosad-treated group on Days 30, 60 and 90, respectively [15]. Flea-free dogs were not assessed in either of those studies. In an earlier study conducted according to a similar protocol, 108 primary dogs were enrolled in a spinosad group, and 46 in a topically applied selamectin group. On Day 90, mean flea count reductions from baseline were 99.9% in the spinosad group and 98.9% in the selamectin group, with 95.4 and 69.6% of dogs, respectively, free of fleas [14]. Another report of 65 primary dogs enrolled in a spinosad-treated group and 63 in a topically-applied fipronil/(S)-methoprene group described Day 90 efficacy of 95.1% for spinosad and 88.4% for fipronil/(S)-methoprene, with 94.8 and 38.2% of dogs free of fleas, respectively [19]. Both studies demonstrated superiority of the orally administered product over the topically applied comparator. Overall, these findings indicate that the performance of lotilaner under field conditions at least matches those reported for other monthly orally administered flea control products.

An important consideration in the development of a product to control fleas and ticks lies in the ease of administration for a dog owner, and both treatments were shown to be palatable for study dogs [20]. Only 6 % of lotilaner treatments were administered directly into a dog’s mouth, with 94% administered free choice, by direct acceptance from the owner’s hand or presented in an empty food bowl, or administered with food. No dogs were withdrawn because of an owner’s inability to administer treatment and all dogs that remained in the study were dosed by their owners according to schedule. The study results therefore confirm the palatability of the lotilaner flavored tablet formulation for dogs.

The low level of gastrointestinal events seen in both groups was similar to that previously reported for antiparasitic products, as were the isolated incidents of abnormal clinical pathology reports that were unrelated to any clinical observations [14, 16, 18, 19, 21]. Under these varied conditions, the absence of treatment-related adverse events confirms the safety of lotilaner in client-owned dogs.

## Conclusion

The results of this study, undertaken in a diverse cohort of client-owned dogs across a broad geographical area of the United States, demonstrate that under a wide range of real-world conditions, lotilaner flavored chewable tablets are easily administered by owners. A single owner-administered lotilaner treatment was greater than 99% effective in reducing mean flea counts by Day 30, the time of the first post-treatment assessment. Three consecutive monthly lotilaner treatments resulted in a 100% reduction in flea infestations, and a substantial reduction in, or elimination of, signs of flea allergy dermatitis. The absence of treatment-related adverse events confirms lotilaner’s safety in dogs. The study therefore demonstrates lotilaner flavored chewable tablets are palatable and that the safety and efficacy of lotilaner are maintained regardless of geography, season and breed of treated dog.

## Additional files

Additional file 1: Spanish translation of the article. (PDF 139 kb)

Additional file 2: French translation of the Abstract. (PDF 43 KB)

## Abbreviations

FAD: Flea allergy dermatitis
FDA-CVM: United States Food and Drug Administration - Center for Veterinary Medicine
SD: Standard deviation
