# Supplementary material for: A randomized, controlled field study to assess the efficacy and safety of lotilaner flavored chewable tablets (Credelio™) in eliminating fleas in client-owned dogs in the USA
Source: Parasit Vectors. 2017 Nov 1;10:528. doi: 10.1186/s13071-017-2469-x (PMC5664423; doi:10.1186/s13071-017-2469-x)
Supplement: Supplementary file 1 — Spanish translation of the article. (PDF 139 kb) [file 13071_2017_2469_MOESM1_ESM.pdf]

Un estudio de campo controlado y aleatorizado para evaluar la eficacia y seguridad de las tabletas masticables saborizadas de lotilaner (Credelio™) para eliminar pulgas en perros con dueño-cliente en EUA

Daniela Karadzovska[1], Kimberly Chappell[2], Shane Coble[2], Martin Murphy[3], Daniela Cavalleri[3], Scott Wiseman[4], Jason Drake[2]* y Steve Nanchen[3]

[1]Elanco Animal Health, Yarrandoo, NSW, Australia
[2]Elanco Animal Health, 2500 Innovation Way, Greenfield, IN 46140, EUA
[3]Elanco Animal Health, Schwarzwaldallee 215, CH-4058 Basilea, WRO-1032.2.58, Suiza
[4]Elanco Animal Health, Basingstoke, Hants, RU

*Correspondencia: drake_jon_j@elanco.com

E-mails:
Daniela Karadzovska: karadzovska_daniela@elanco.com
Kimberly Chappell: chappell_kim@elanco.com
Shane Coble: shane.coble@elanco.com
Martin Murphy: murphy_martin_gerard@elanco.com
Daniela Cavalleri: cavalleri_daniela_a@elanco.com
Scott Wiseman: wiseman_scott@elanco.com
Jason Drake: drake_jon_j@elanco.com
Steve Nanchen: nanchen_steve@elanco.com

Resumen

Antecedentes: Los estudios preclínicos han demostrado que la nueva isoxazolina, lotilaner (Credelio™, Elanco) administrada oralmente a perros, produce una rápida fulminación de pulgas y garrapatas y una velocidad para matar sostenida por al menos un mes post-tratamiento con un amplio margen de seguridad. Un estudio de campo se llevó a cabo para validar los resultados pre-clínicos.

Métodos: Los perros se enrolaron en 10 clínicas veterinarias a lo largo de Estados Unidos. Los hogares que calificaron contenían hasta tres perros y un perro primario con al menos 10 pulgas se aleatorizaron 2:1 para recibir lotilaner (Credelio™, Elanco) a la dosis mínima recomendada de 20 mg/kg, o afoxolaner (Nexgard®, Merial), administrado de acuerdo a las indicaciones de la etiqueta, para proporcionar una dosis mínima de 2.5 mg/kg. Los tratamientos se dispensaron en los Días 0, 30 y 60 para que fueran administrados por los dueños; todos los perros de los hogares recibieron el mismo tratamiento que el perro primario. Los conteos de pulgas y garrapatas post-enrolamiento se hicieron en los perros primarios en los Días 30, 60 y 90, y en todos los perros se evaluó la palatabilidad de la tableta y la seguridad.
Resultados: Para las evaluaciones de eficacia, se utilizaron datos de 111 perros tratados con lotilaner y 50 perros tratados con afoxolaner; para la seguridad 197 y 86 perros, respectivamente. Las reducciones porcentuales de la base en la media geométrica de los conteos de pulgas para el grupo de lotilaner fueron de 99.3, 99.9 y 100% en los Días 30, 60 y 90, respectivamente, y para afoxolaner 98.3, 99.8 y 99.8% ($P < 0.001$, ambos grupos, todos los días). En el Día 90, 100% de los perros tratados con lotilaner y 93% en los perros tratados con afoxolaner estaban libres de pulgas. Muy pocas garrapatas estuvieron presentes para permitir la evaluación. No hubo diferencias en palatabilidad entre los productos ($P = 0.2132$), con un 94% y 96% de los tratamientos de lotilaner y afoxolaner, respectivamente, siendo aceptados cuando se ofrecieron en la mano, en un tazón de alimento vacío o con alimento. Ambos tratamientos fueron bien tolerados, aliviando los signos clínicos de la dermatitis alérgica por pulgas (DAP) en perros afectados en el momento de enrolarlos.
Conclusión: Un solo tratamiento de lotilaner administrado por el dueño fue mayor que el 99% efectivo al reducir los conteos medios de pulgas al cabo de 30 días. Tres tratamientos consecutivos mensuales de lotilaner resultaron en un 100% de reducción en las infestaciones de pulgas, y una reducción sustancial en los signos de DAP. Las tabletas saborizadas de lotilaner fueron fácilmente aceptadas bajo condiciones de campo. La ausencia de eventos adversos relacionados con el tratamiento confirma la seguridad de lotilaner en perros.
Palabras clave: Credelio, Lotilaner, Pulgas, Afoxolaner, Perros, Estudio de campo

Antecedentes

El prurito es un resultado común de las infestaciones de pulgas en perros y puede progresar en alopecia y condiciones dermatológicas más severas, incluyendo la dermatitis alérgica por piquete de

pulga (DAP) [1]. En algunos perros, la inyección de la saliva de la pulga cuando el parásito se alimenta puede llevar a un incremento general en la carga antigénica y resultar en erupciones atópicas en perros susceptibles [2]. Se ha demostrado que las pulgas son vectores de enfermedades zoonóticas, tales como aquellas causadas por *Rickettsia* spp. y *Bartonella* spp., y son hospedadores intermedios de la tenia *Dipylidium caninum* que puede desarrollarse hasta etapas adultas en niños que ingieren pulgas infectadas [3, 4]. En las infestaciones de pulgas no controladas, la contaminación de las premisas del hogar también puede llevar a problemas por el piquete de la pulga en humanos expuestos [4].

La pulga hembra es una prolífica ponedora de huevos, y bajo condiciones de laboratorio se ha demostrado que comienza a poner huevos al cabo de 24 a 36 horas después de encontrar al hospedador, y luego pone tantos como 50 huevos por día, con una producción de huevos diaria que continúa durante toda la vida de más de 100 días [5]. Los huevos de pulga se caen del animal hospedador y luego proveen el potencial para un enorme incremento en las etapas inmaduras del ciclo de vida en el ambiente. En ausencia de tratamientos efectivos estas etapas continúan desarrollándose para presentar un aumento en el desafío de la pulga. El uso de plaguicidas ambientales para eliminar la población de pulgas del hogar puede no ser efectivo, y también pone en riesgo a los habitantes del hogar a la exposición al plaguicida [6]. Por lo tanto, es importante que los animales infestados sean tratados con productos que eliminen las cargas de pulgas existentes y provean protección contra los desafíos post-tratamiento por un ambiente contaminado.

La nueva isoxazolina, lotilaner, provee a los veterinarios y a sus clientes una medida efectiva de rápida acción y duradera para el control de las infestaciones por pulgas y garrapatas en los perros. Se ha demostrado en estudios de seguridad y eficacia que lotilaner es bien tolerado, incluyendo en un estudio en cachorros de ocho semanas de edad al inicio del tratamiento, en donde la dosis administrada era de hasta 215 mg/kg, un día por mes (cada dosis diaria más de 10 veces la dosis mínima recomendada) durante tres meses [7-11]. En estudios de laboratorio, se demostró que lotilaner comienza a matar pulgas y garrapatas al cabo de cuatro horas después del tratamiento [8, 9]. La eficacia contra las pulgas y garrapatas, luego fue sostenido hasta 35 días después del tratamiento, indicando que el uso mensual de lotilaner será efectivo al causar la disminución de las etapas del ciclo de vida de la pulga del ambiente del perro [10, 11].

Se diseñó un estudio de campo para confirmar los resultados de los estudios de desarrollo pre-clínico. El objetivo primario del estudio fue evaluar la eficacia y la seguridad de las tabletas masticables, saborizadas de lotilaner administradas oralmente por los dueños de los perros a un objetivo de nivel mínimo de dosis de 20 mg/kg para el tratamiento y el control de las infestaciones

de pulgas. Los objetivos secundarios fueron evaluar la presencia y la persistencia de los signos clínicos asociados con la DAP (prurito, eritema, descamación, pápulas, alopecia, dermatitis/piodermatitis), para evaluar la aceptación de la formulación, y en el evento de una infestación por garrapata, evaluar la actividad contra las garrapatas en perros infestados naturalmente.

Métodos

Este fue un estudio de campo aleatorizado, doble ciego, con control positive con perros enrolados en clínicas veterinarias en todo Estados Unidos. El protocolo se preparó en cumplimiento con los lineamientos de la Asociación Mundial para los Avances en Parasitología Veterinaria (WAAVP) para evaluar la eficacia de los parasiticidas para el tratamiento, prevención y control de la infestación de pulgas y garrapatas en perros y gatos [12]. El estudio se condujo y se documento de acuerdo con el Código Federal de Regulaciones de EUA, Título 21, Parte 511, Sección 511.1, *Nuevos Fármacos para Animales para Uso en Investigación Exentos de la Sección 512(a) de la Ley* (Abril 2013) y de la Guía para la Industria 85 de la Administración de Alimentos y Fármacos de los Estados Unidos – Centro para Medicina Veterinaria (FDA/CVM), Cooperación Internacional para la Armonización de los Requisitos Técnicos para el Registro de los Productos Veterinarios (VICH) GL9, *Buenas Prácticas Clínicas* (Mayo 2001).

Animales y hogares

Para que un hogar fuera enrolado en el estudio se requería que tuvieran al menos uno y no más de tres perros, todos deberían de tener al menos ocho semanas de edad, pesar al menos dos kilogramos, y estar clínicamente sanos o tener enfermedades menores que a juicio no interfirieran en el estudio. Los perros con enfermedades crónicas (i.e. diabetes, hipotiroidismo, osteoartritis) se consideraron estables o controlados eran candidatos para ser enrolados. Se requirió que al menos un perro en el hogar tuviera 10 pulgas.

Algunos hogares fueron excluidos por las siguientes razones: si contenían perros que fueran a ser utilizados para reproducción, o que estuvieran gestantes o en lactancia y si hubo algún tratamiento ambiental contra pulgas en los tres meses previos al estudio. Un hogar también quedaría excluido del estudio si contenía perros en un tratamiento concurrente que posiblemente interfiriera con el comportamiento o la interpretación de los resultados del estudio (e.g. tratamiento con otra

preparación ectoparasiticida). El tiempo mínimo para el retiro de dichos tratamientos corresponde a la duración de la eficacia presentada en la etiqueta. Sí el producto aplicado no se identificaba claramente, el tiempo mínimo de retiro fue de cuatro semanas. Sí el tratamiento aplicado era un collar, el tiempo mínimo de retiro era de dos semanas previo a la participación en el estudio. Para evitar cualquier factor potencial de confusión que pudiera haber impactado el conteo de pulgas en el Día 0, se hizo una exclusión por baño/aplicación de shampoo en perros del estudio al cabo de 48 horas previas al tratamiento.

A parte de esa restricción pretratamiento, o el uso de cualquier producto activo contra pulgas y/o garrapatas, no hubieron restricciones en mojar o bañar a los perros, y tampoco hubieron restricciones por la presencia de mascotas no caninas en el hogar. No hubo estipulación acerca de mantener a los perros dentro o fuera de casa. Los gatos presentes en los hogares del estudio fueron tratados una vez al mes, a lo largo de la duración del estudio, con un adulticida para pulgas comercialmente disponible que fue dispensado en las clínicas al momento de dispensar los tratamientos para el estudio.

La unidad experimental fue el perro primario en cada hogar. Sí más de un perro en un hogar cumplía con todos los criterios de inclusión, incluyendo la carga de al menos 10 pulgas, se seleccionaba el primer perro, de todos los perros que calificaban, por el orden alfabético de los nombres, y este sería el perro primario del hogar. Todos los perros en el hogar que recibieron el tratamiento regresaban al menos a una visita de seguimiento y fueron incluidos en los análisis de seguridad. Los perros tenían dueño y por lo tanto, eran alimentados, alojados y manejados por sus dueños. Los procedimientos veterinarios estándar fueron llevados a cabo por cada clínica. Se le solicitó a los dueños que mantuvieran a los perros del estudio en la misma dieta a lo largo del estudio.

Los perros podían ser retirados del estudio a discreción del investigador, sí un dueño retiraba el consentimiento, o por cualquier evento adverso que requiriera detener el tratamiento del estudio u observaciones. Otras razones para el retiro incluían la administración de cualquier tratamiento concomitantes de un protocolo prohibido, la falta de eficacia de cualquier producto, la pérdida de del hogar para el seguimiento, y desviaciones del protocolo que pudieran haber comprometido la integridad del estudio.

Enrolamiento

En cada clínica, los perros del estudio se pesaron, y recibieron un examen físico incluyendo la calificación de la condición corporal y, para cualquier perro con al menos 10 pulgas se le hicieron

evaluaciones de signos de DAP. Se colectaron muestras de sangre de todos los perros del estudio para pruebas de hematología y química sanguínea, y también se colectó orina para el urianálisis, como una evaluación base de salud general. Todos los perros fueron peinados para realizar los conteos de pulgas y garrapatas. Cuando todos los perros en un hogar se encontraron como candidatos, con al menos un perro con 10 o más pulgas, el hogar se enrolaba en el estudio. Dentro de cada clínica, los perros primarios se colocaron en bloques de tres de acuerdo al orden del enrolamiento del hogar y aleatoriamente se ubicaron a un grupo de tratamiento en una proporción de 2:1 de lotilaner a afoxolaner. Los otros perros en el hogar, a un máximo de dos perros suplementarios por hogar, recibieron los mismos tratamientos de acuerdo al mismo calendario como los perros primarios del hogar. Los perros suplementarios no se evaluaron en las visitas subsecuentes para conteos de pulgas y garrapatas, pero se evaluaron para DAP, si su conteo de pulgas en la visita de enrolamiento era de al menos 10, y si los signos clínicos de la DAP estaban presentes en la visita de enrolamiento. No se hicieron evaluaciones posteriores de DAP después del Día 0 si no habían signos en la visita de enrolamiento. El enrolamiento objetivo del estudio fue de 100 perros primarios para lotilaner y 50 perros primarios para afoxolaner.

Tratamientos

Los tratamientos despachados a los dueños para su administración en casa fueron:

(i) Lotilaner (Credelio™, Elanco, Greenfield, IN, EUA) disponible en cada clínica para dispensar en cuatro tamaños de tableta: 56.25 mg, 112.5 mg, 225 mg y 450 mg, a ser administradas con base al peso corporal de cada perro en el hogar, a un nivel mínimo de dosis recomendada de 20 mg/kg.

(ii) Afoxolaner (Nexgard®, Merial, Duluth, GA, EUA) disponible para cada clínica para dispensar en cuatro tamaños de tabletas diferentes: 11.3 mg, 28.3 mg, 68 mg y 136 mg, a ser administradas de acuerdo a las indicaciones de la etiqueta a una dosis mínima de 2.5 mg/kg.

En cada clínica, el veterinario encargado de examinar y conducir el examen físico general, y evaluar la DAP y la calificación de condición corporal era ciego a los tratamientos. La persona(s) despachando los tratamientos del estudio a los dueños era responsable para la ubicación del grupo de tratamiento, entrenar al dueño acerca del tratamiento de los animales, y la responsabilidad del fármaco. No se divulgó información relacionada con el tratamiento al veterinario examinador (y/o a los asignados a entrenar), y los registros se mantuvieron por separado de los registros del veterinario examinador. En los blisters individualizados se colocaron etiquetas para ocultar toda información, cualquier texto existente para que los dueños permanecieran ciegos al tratamiento. En la visita

inicial y en la segunda y tercera visitas, la persona que dispensaba el tratamiento en cada clínica proporcionó un número adecuado de tabletas para cada perro del hogar a ser tratado una vez cada uno de los Días 0, 30 (± 2), y 60 (± 2). Se les dieron instrucciones a los dueños para alimentar a sus perros aproximadamente 30 minutos previo al tratamiento.

Cada dueño de perro fue instruido para que inicialmente ofreciera la tableta de su mano por aproximadamente 90 segundos. Sí el perro no la aceptaba y consumía la tableta de la mano, la tableta se tenía que colocar en el tazón vacío del perro por aproximadamente 90 segundos. Sí el perro seguía sin comer la tableta, se le ofrecía una pequeña cantidad de alimento por aproximadamente 90 segundos. Sí esto no tenía éxito, el dueño tendría que administrar la tableta directamente en la boca del perro, en la parte trasera de la lengua, y luego alentar al perro a tragarla. Sí la tableta era vomitada al cabo de 60 minutos de su administración, el dueño tenía que contactar al investigador para que se le proporcionara un reemplazo.

Se permitieron los tratamientos concomitantes mientras que no interfirieran con los objetivos del estudio. Algunas medicaciones concomitantes, tales como corticosteroides, antihistamínicos y antibióticos administrados para los signos de DAP, requirieron la exclusión de los datos de evaluación de la DAP. Los productos administrados /dispensados de manera rutinaria tales como las vacunaciones, los preventivos del gusano de corazón, los parasiticidas intestinales o los suplementos nutricionales se aceptaron.

Conteo de pulgas y garrapatas y la evaluación de la dermatitis alérgica por pulgas

Se llevaron a cabo conteos de pulgas con el peine en los Días 0, 30, 60 y 90 para el perro primario en cada hogar. En cada visita, el número de garrapatas encontradas durante el peinado también se registró. Se contaron manualmente las pulgas y las garrapatas peinando todo el cuerpo de cada perro por aproximadamente 20 minutos usando un peine con dientes finos. En la visita inicial, sí menos de 10 pulgas se contaban al cabo de los 5 minutos iniciales del peinado, el conteo se detenía. Las infestaciones de más de 250 pulgas se registraron como > 250, y para tales perros fuertemente infestados, el valor de 251 se usó para el análisis. Las pulgas que se peinaron se desecharon y no se regresaron a ningún perro.

El veterinario examinador también evaluó a cada perro de estudio con un conteo de pulgas de al menos 10 pulgas, en el Día 0 y (± 2 días) Días 30, 60 y 90 para signos de DAP, clasificando cada signo (prurito, pápulas, eritema, alopecia, descamación, dermatitis/ piodermatitis) como Ausente, Leve, Moderado o Severo. En donde fue posible el mismo veterinario completó cada evaluación de seguimiento para cada perro primario y suplementario en los que estuviera presente

cualquier signo de DAP en la visita de enrolamiento. Para los perros sin signos clínicos asociados con la DAP en el Día 0, no se condujeron evaluaciones de DAP adicionales en los Días 30, 60 y 90, pero la aparición subsecuente de dichos signos se registró como un evento adverso.

Evaluaciones y estadística

La eficacia de cada tratamiento en el control de la infestación por pulgas fue evaluado comparando la línea base de los conteos en el Día 0 con aquellos en los días 30, 60 y 90 después de la visita de enrolamiento. La eficacia se determine con base al porcentaje de reducción en los conteos de pulgas adultas en la pre- a post-dosificación dentro de cada grupo de tratamiento. El porcentaje de eficacia en cada punto de tiempo del conteo se calculó como sigue a continuación:

Porcentaje de eficacia = ([MB – MA]/ MB) × 100, en donde MB es la media del conteo de las pulgas previo a la dosificación (Día 0) y MA es la media del conteo de las pulgas post-dosificación (Día 30, 60 y 90).

Los cálculos se completaron usando las medias geométricas para la determinación de la eficacia, y también se calcularon las medias aritméticas. El cálculo de las medias geométricas involucró el tomar el logaritmo del conteo de pulgas de cada perro. Sí cualquiera de los conteos de pulgas era igual a cero, se agregó un uno al conteo para cada animal en el grupo y luego se restó de la media resultante previo al cálculo del porcentaje de eficacia.

Para cada grupo de tratamiento, los datos de pulgas transformados-logarítmicamente (conteo + i) se analizaron pre- a post-dosificación (procedimiento SAS PROC MIXED) para determinar si la reducción en el conteo de pulgas era estadísticamente significativo de la línea base a lo ocurrido en cada punto de tiempo. El modelo incluyó un efecto fijo *Pareado* que se definió como un indicador variable (0, 1; para representar los conteos pre- y post-dosificación), y también incluyó los efectos aleatorios del Sitio. Se adecuaron modelos separados para cada uno de los Días 30, 60 y 90 en comparación con el Día 0. Cualquier tratamiento se consideró efectivo en cada punto del tiempo si se cumplía con el siguiente criterio: (i) Los animales se infestaron adecuadamente con las pulgas previo a la dosificación (≥ 10 pulgas); (ii) La eficacia calculada en el punto de tiempo fue ≥ 90%; (iii) Hubo un decremento estadísticamente significativo un nivel de significancia de 2-lados de 0.05 ($P < 0.05$) en los conteos de pulgas pre- a post-dosificación en el punto en el tiempo.

Este estudio fue diseñado para cumplir con los requerimientos regulatorios, y las comparaciones entre-grupo no fueron objetivo del estudio. Sin embargo, las comparaciones con el

grupo de tratamiento se completaron en cada punto en el tiempo con respecto al conteo de pulgas y la proporción de perros libres de pulgas. Se adecuaron modelos separados para cada uno de los Días 30, 60 y 90. Para comparar los conteos de pulgas, los datos transformados logarítmicamente (conteo + 1) se analizaron (procedimiento SAS PROC MIXED) con el grupo de tratamiento y el conteo de pulgas de la línea base como efectos fijos y el sitio como efecto aleatorio. La proporción de perros con cero pulgas se comparó entre los dos grupos de tratamiento usando la prueba exacta de Fisher.

Una calificación total de DAP se calculó para cada animal en cada punto de tiempo como la suma de las calificaciones de los signos clínicos (calificaciones de prurito, eritema, descamación, pápulas, alopecia, dermatitis/piodermatitis) y se evaluaron sobre tiempo usando el procedimiento de SAS PROC MIXED. Las diferencias dentro del grupo de tratamiento sobre el tiempo se determinaron a través de la declaración de LSMEANS.

Para la evaluación de la palatabilidad relativa de cada producto de estudio, se adecuó un modelo mixto generalizado de medidas repetidas. El modelo utiliza una distribución de respuesta binomial y la función del enlace *logit.* La palatabilidad de la tableta (aceptada/ no aceptada) fue la respuesta variable. El grupo de tratamiento, día de estudio (Día 0, 30, 60) y el grupo de tratamiento por la interacción del día del estudio fueron efectos fijos. Puesto que cada perro se dosificó en múltiples ocasiones la correlación entre las observaciones sucesivas en el mismo perros se incorporaron al modelo. El mayor efecto en el grupo de tratamiento se utiliza para probar la hipótesis nula donde no hay diferencia en la tasa de aceptación de las tabletas a lo largo de toda la duración del estudio entre los grupos de tratamiento. Los intervalos de confianza para la tasa de aceptación también fueron calculados.

Resultados

De Julio a Diciembre, 2014, 122 perros primarios se enrolaron en el grupo de lotilaner (en total 214, incluyendo a los perros suplementarios en el hogar) y 58 perros primarios en el grupo de afoxolaner (98 perros en total) de 10 clínicas de pequeñas especies a lo largo de todo Estados Unidos (una clínica en California, Florida, Georgia, Louisiana, Michigan, Missouri, Oregon, Pennsylvania, Carolina del Sur y Texas). Para la población de seguridad, se definió como perros enrolados que recibieron al menos un tratamiento del estudio y regresaron al menos para una visita post-tratamiento, siendo 197 y 86 perros en el grupo de lotilaner y afoxolaner, respectivamente. La media de la edad de los perros en la población de seguridad de ambos grupos fue aproximadamente de 5.5

años (Tabla 1). Hubo una distribución similar de los grupos de edad, y aproximadamente el 82% de los perros en cada grupo era mayor a los 12 meses. Las edades mínimas eran 2 meses en el grupo de lotilaner y 3 meses en el grupo de afoxolaner, y los pesos mínimos eran de 2.0 kg y 2.2 kg, respectivamente. El sexo y el estado de esterilización de los perros enrolados fue similar en ambos grupos, así como lo fue la distribución de hogares con un solo perro o varios. Perros de raza pura comprendían un 53.8% de los perros enrolados en el grupo de lotilaner (36 razas diferentes) y 61.6% de los perros en el grupo de afoxolaner (22 razas diferentes), siendo que los Chihuahuas y los Labrador Retrievers eran las razas más frecuentemente enroladas en cada grupo.

De los 312 perros primarios y suplementarios que se enrolaron, 259 completaron el estudio (179 en los grupos de hogares de lotilaner y 80 en los grupos de hogares de afoxolaner). De los 312, 53 perros (35 lotilaner; 18 afoxolaner) terminaron prematuramente. Para las evaluaciones de eficacia, se utilizaron datos de 111 perros tratados con lotilaner y 50 perros tratados con afoxolaner. Los grupos de análisis y casos excluidos se definieron en colaboración con la FDA-CVM. Las razones para la exclusión incluyeron la pérdida del seguimiento, la presentación de un evento adverso que requiriera detener las observaciones, el retiro del consentimiento del dueño y la muerte (un perro primario y un perro secundario murieron, esto se discute a continuación).

Ambos grupos mostraron reducciones estadísticamente significativas ($P < 0.001$; Tabla 2) en la media de los conteos de pulgas de la línea base (pre-tratamiento, visita Día 0) al final del estudio. En este punto el 100% de los perros tratados con lotilaner y el 93% de los tratados con afoxolaner estaba libre de pulgas (Fig. 1). Esta diferencia en proporciones de perros libres de pulgas entre los dos grupos de tratamiento fue estadísticamente significativa ($P = 0.0323$, cuadro de probabilidad = 0.0323) (Tabla 3). La comparación estadística de los grupos de tratamiento también demostró que hubo significativamente menos pulgas en los perros en el grupo de lotilaner que en el grupo de afoxolaner en el Día 30 ($t_{(143)} = 2.63$, $P = 0.0095$) y 90 ($t_{(125)} = 2.37$, $P = 0.0193$). El porcentaje de las reducciones geométricas en la media del conteo de pulgas para el grupo de lotilaner fue de al menos un 99% en los Días 30, 60 y 90, y para el grupo de afoxolaner fue mayor del 99% sólo en los Días 60 y 90 (Tabla 2).

Muy pocos perros presentaron garrapatas como para hacer una evaluación del estudio válido para la eficacia. Por lo tanto, no hubo un análisis del conteo de garrapatas.

Ambos productos fueron bien aceptados cuando se administraron por los dueños de los perros. Para las tabletas de lotilaner, el 94% fueron aceptadas cuando se ofrecieron a mano, en un tazón de alimento vacío o con alimento. Solamente, el 6% de los perros en el grupo de lotilaner y 4% en el grupo de afoxolaner recibió la tableta directamente en la boca, y el 100% de los

tratamientos fue exitosamente administrado en ambos grupos. No hubo efectos significativos para el grupo de Tratamiento ($F_{(1,281)}$ = 1.56, P = 0.2132), Día del estudio ($F_{(2,524)}$ = 1.41, P = 0.2458) o la interacción entre el grupo y el día del estudio ($F_{(2,524)}$ = 0.01, P = 0.9885). No se reportaron perros que hubieran vomitado al cabo de una hora después de recibir el tratamiento, y ningún perro requirió la redosificación con cualquiera de los productos.

Entre los Días 0 y 30, 26 (12.1%) perros del grupo de lotilaner y 19 (19.4%) perros del grupo de afoxolaner recibieron tratamiento para la DAP y por lo tanto fueron inelegibles para continuar con las evaluaciones de la DAP. Hubieron 55 perros en el grupo de lotilaner y 29 en el grupo de afoxolaner en los cuales los signos clínicos de DAP (prurito, pápulas, eritema, alopecia, descamación, dermatitis/piodermatitis) se presentaron en la línea base y luego se siguieron a lo largo del estudio. En la línea base, el prurito y el eritema fueron, en conjunto, los signos más prevalentes de DAP observados en el grupo de lotilaner (41 perros con cada signo, i.e. 33.6% de los perros primarios enrolados), con un perro registrado con signos severos de prurito. Se observó una mejoría en 40 de 41 perros durante el curso del estudio y para el Día 90 los signos más severos se registraron solamente como leves. El prurito y el eritema también fueron los signos más prominentes en el grupo de afoxolaner, afectando a 24 (41.4%) y 21 (36.2%) perros en el Día 0, respectivamente. Una reducción significativa en la presencia general y persistencia de los signos de DAP se presentaron en ambos grupos de tratamiento para el Día 30 (lotilaner $t_{(129)}$ = 9.79, P < 0.001; afoxolaner $t_{(131)}$ = 6.67, P < 0.001), cuando 52 (94.5% de los perros mostraron signos en la línea base) de los perros en el grupo de lotilaner y 25 (86.2%) en el grupo de afoxolaner mostraron una mejoría. Para el Día 90, los signos de DAP se habían resuelto o mejorado y permanecieron significativamente más bajos que la línea base en el Día 60 (lotilaner $t_{(142)}$ = 10.46, P < 0.001; afoxolaner $t_{(141)}$ = 7.98, P < 0.001) y 90 (lotilaner $t_{(142)}$ = 10.47, P < 0.001; afoxolaner $t_{(141)}$ = 8.14, P < 0.001).

El porcentaje de perros del grupo de lotilaner con al menos un evento adverso reportado sobre los tres períodos de estudio, Día 0–30, Día 30–60, y Día 60–90, fue 10.7, 8.1 y 9.1%, respectivamente, comparado a 16.3, 12.9 y 3.8% en perros que recibieron afoxolaner. A lo largo de los tres meses del estudio, se observaron eventos adversos en 24.4% de los perros del grupo de lotilaner (13.6% de las dosis administradas) y 26.7% de los perros del grupo de afoxolaner (16.5% de las dosis administradas). La mayoría de estos eventos se clasificaron como Desórdenes en Piel y Apéndices (principalmente relacionados con la dermatitis) observados en un 7.1% de los perros tratados con lotilaner y un 9.3% de los perros tratados con afoxolaner. Cierto número de perros en cada grupo mostró eventos aislados de cambios de apetito y letargia, además de edema localizado reportado en unos perros que recibieron afoxolaner. Las observaciones del tracto digestivo,

incluyendo vómito y diarrea, ocurrieron en un nivel bajo en cada grupo, involucrando a un 5.1% de los perros tratados con lotilaner y 7.0% de aquellos que recibieron afoxolaner. La tasa de incidencia de los eventos adversos, incluyendo los eventos adversos que involucraban dermatitis, disminuyó en ambos grupos del Día 0 a la evaluación final. Hubo diferencias significativas en ambos grupos entre la línea base y el Día 90 de la salida del estudio para los índices de varios parámetros de hematología, química clínica y urianálisis. Sin embargo, todas las medias aritméticas permanecieron dentro de los rangos normales, y mientras que algunos valores cayeron fuera de los rangos de referencia de la patología clínica, no fueron considerados clínicamente relevantes por los investigadores del estudio.

El perro primario que murió era un Yorkshire Terrier de siete años de edad en el grupo de lotilaner que tuvo signos radiológicos de cardiomegalia y un diagnóstico de enfermedad pulmonar obstructiva crónica en el enrolamiento. La condición de este perro se deterioró y en la necropsia post-eutanasia se encontró un histiocitoma esplénico maligno. El perro secundario que murió, también en el grupo de lotilaner era un Pomeranian de 13 años de edad que entró en una afección respiratoria a la mitad del tiempo entre la segunda y la tercer visita. En el enrolamiento, este perro tuvo una enfermedad periodontal severa combinada con proteinuria 3+ y nitrógeno ureico en sangre/creatina (BUN/Cr) elevado, sugiriendo una enfermedad renal subyacente. El dueño reporte la muerte de le perro dos semanas después del reporte de la afección respiratoria y no fue posible dar seguimiento. Ninguno de los eventos en estos dos perros se consideró que estuviera relacionado con el tratamiento.

En el grupo de afoxolaner, un perro primario, un Border Collie/cruzado con Labrador, sufrió dos convulsiones de leves a moderadas, cada una aproximadamente un mes después de recibir el tratamiento del estudio programado. Este perro permaneció en el estudio. El evento adverso que resultó en el retiro de un perro tratado con afoxolaner fue un incidente de letargia, vómito y diarrea del cual el perro se recuperó y el cuál el investigador lo consideró no relacionado con el tratamiento. Otros eventos adversos severos que se presentaron en los perros del estudio incluyeron los desórdenes renal y urinario, absceso y desórdenes del tracto digestivo (uno en cada grupo). Los respectivos investigadores consideraron que la relación de estos desórdenes con los tratamientos del estudio eran desconocidas o poco probables, y ninguno de los retiros fue atribuido al tratamiento.

Un rango de medicaciones concomitantes se administró a los perros tratados con lotilaner, incluyendo aquellas no autorizadas por el protocolo, que iban desde butorfanol, clorhidrato de ketamina, clases múltiples de antibióticos, anticonvulsivos, fármacos antiinflamatorios no esteroidales, oclacitinib, corticosteroides, macrólidos y antihelmínticos de benzimidazol y productos

óticos. Las vacunas contra la rabia, y multivalentes se administraron a más de un 10% de perros tratados con lotilaner y todos los tratamientos concomitantes parecieron ser bien tolerados.

Discusión

Los diversos antecedentes genéticos de los perros enrolados en el grupo de lotilaner (36 razas además de los perros criollos), y su amplio espectro geográfico a lo largo de las diferentes regiones de los Estados Unidos proveen una sólida representación del mundo real en el que un producto antiparasitario será usado. Más aún, los tiempos del estudio del verano a los inicios de la primavera significa que los hogares del estudio y los perros estarían expuestos a los factores estacionales que pueden precipitar las condiciones dermatológicas, y pueden incrementar la exposición a las infestaciones por pulgas [2, 13]. Independientemente de las condiciones de campo, los resultados se alinean con los estudios de laboratorio que indican que lotilaner es un tratamiento para el control de pulgas seguro y efectivo [7, 8, 10]. Tanto lotilaner, como afoxolaner demostraron ser altamente efectivos a partir del primer tratamiento en adelante. En cada evaluación post-Día 0, la media de los conteos de pulgas, el porcentaje de los perros infestados y el conteo máximo de pulgas en un perro individualmente eran consistente y numéricamente más bajos en el grupo de lotilaner que en el grupo de afoxolaner. Al final del estudio no se encontraron pulgas en ningún perro tratado con lotilaner.

La calificación de lesiones de la DAP como leve, moderada y severa, que se usó en este estudio, es consistente con lo que se ha descrito para otros estudios de campo que evalúan la efectividad de los productos introductorios para el control de pulgas [14–16]. Una limitante de esta metodología en la calificación es que no ha sido validada y es subjetiva, entonces el grado de severidad puede variar entre los clínicos. Sin embargo, como con otros reportes, la mejoría progresiva y marcada en cada signo clínico de la DAP en los perros tratados puede ser atribuida a dos factores relacionados con el rápido inicio y velocidad residual sostenida para matar de lotilaner [8, 10]. Un factor es reducir el desafío antigénico que resulta de la rápida fulminación de las recién emergidas pulgas. Relacionado con la fulminación rápida, el segundo factor se basa en la velocidad sostenida para matar a las pulgas, que elimina a las pulgas recién emergidas del perro antes de que comience la ovoposición, por ende permitiendo una disminución progresiva de la biomasa de pulgas en el hogar, llevando a su completa eliminación.

Los resultados en este estudio se alinean con aquellos reportados en un estudio de campo europeo, conducido de acuerdo a un protocolo similar, en el que la eficacia de lotilaner en 128 perros primarios fue del 99.5, 99.9 y 99.8% en los Días 28, 56 y 84, respectivamente, con un 98.4% de los perros tratados libres de pulgas en el Día 84 [17]. Se utilizó un protocolo similar para investigar la eficacia de otros productos de control de pulgas que se administran oralmente cada mes. Un estudio en los EUA de otro compuesto de las isoxazolinas, sarolaner, comparó los resultados de 195 perros primarios tratados con sarolaner y 98 con spinosad. La eficacia de ambos productos fue del > 99% en los Días 60 y 90 [18]. También un estudio europeo comparó el desempeño de sarolaner con spinosad, enrolando 93 y 44 perros en cada grupo, respectivamente. La eficacia reportada fue del 99.4, > 99.9 y > 99.9% en el grupo tratado con sarolaner y del 93.7, 96.8 y 95.1% en el grupo tratado con spinosad en los Días 30, 60 y 90, respectivamente [15]. Los perros libres de pulgas no se evaluaron en ninguno de esos estudios. En un estudio anterior conducido de acuerdo a un protocolo similar, 108 perros primarios se enrolaron en un grupo de spinosad, y 46 en un grupo con selamectina tópica. En el Día 90, la media de las reducciones en el conteo de pulgas a partir de la línea base fue del 99.9% en el grupo de spinosad y 98.9% en el grupo de selamectina con 95.4 y 69.6% de los perros libres de pulgas, respectivamente [14]. En otro reporte de 65 perros primarios enrolados en el grupo tratado con spinosad y 63 en el grupo de fipronil/(S)-metopreno aplicado tópicamente se describió en el Día 90 una eficacia del 95.1% para spinosad y 88.4% para fipronil/(S)-metopreno, con 94.8 y 38.2% de los perros libres de pulgas, respectivamente [19]. Ambos estudios demostraron la superioridad del producto administrado oralmente sobre el comparativo aplicado tópicamente. En general, estos hallazgos indican que el desempeño de lotilaner bajo condiciones de campo, al menos, coincide con aquellos reportados para otros productos para el control de pulgas administrados oral y mensualmente.

Una consideración importante en el desarrollo del producto para el control de pulgas y garrapatas recae en la fácil administración por parte del dueño del perro, y se demostró que ambos tratamientos fueron palatables para los perros del estudio [20]. Sólo el seis porciento de los tratamientos de lotilaner se administraron directamente en la boca del perro, con un 94% administrado a libre acceso, por aceptación directa de la mano del dueño o presentado en el tazón de alimento vacío, o administrado con el alimento. Ningún perro fue retirado del estudio debido a la inhabilidad del dueño para administrar el tratamiento y todos los perros que permanecieron en el estudio fueron dosificados por sus dueños de acuerdo al calendario. Por lo tanto, los resultados del estudio confirman la palatabilidad de la formulación de lotilaner en tableta saborizada para perros.

El bajo nivel de eventos gastrointestinales visto en ambos grupos fue similar al previamente reportado para otros productos antiparasitarios, así como los incidentes aislados de reportes de patología clínica anormal que no se relacionaron con ninguna observación clínica [14, 16, 18, 19, 21]. Bajo estas condiciones variadas, la ausencia de eventos adversos relacionados con el tratamiento confirma la seguridad de lotilaner en perros con dueño.

Conclusión

Los resultados de este estudio, llevado a cabo en una cohorte diversa de perros con dueño a lo largo de una amplia área geográfica de los Estados Unidos, demostraron que bajo un amplio rango de condiciones en el mundo real, lotilaner en tabletas masticables saborizadas son fácilmente administradas por los dueños. Un solo tratamiento con lotilaner administrado por el dueño tuvo más de un 99% de efectividad al reducir la media de los conteos de pulgas para el Día 30, el tiempo de la primera evaluación post-tratamiento. Con tres tratamientos consecutivos mensuales con lotilaner se obtuvo un resultado del 100% en la reducción de las infestaciones por pulgas, y una reducción substancial en, o eliminación de los signos de la dermatitis alérgica por pulgas. La ausencia del eventos adversos relacionados con el tratamiento confirman la seguridad de lotilaner en perros. Por lo tanto, el estudio demostró que lotilaner en tabletas masticables saborizadas es palatable y que la seguridad y eficacia se mantuvieron, independientemente, de la geografía, la estación del año y la raza del perro tratado.

Abreviaturas

DAP: dermatitis alérgica por pulgas; FDA-CVM: Administración de Alimentos y Fármacos de los Estados Unidos – Centro para la Medicina Veterinaria; DE: desviación estándar

Reconocimientos

Los autores quieren agradecer al Dr. Bill Ryan de Ryan Mitchell Associates LLC por su apoyo con el manuscrito.

Aprobación de ética y consentimiento para participar

El estudio fue aprobado por el Grupo de Bienestar Animal de Novartis Salud Animal Global. A los dueños de perros participantes se les solicitó que firmaran una forma de consentimiento con

información para que su perro(s) participaran en el estudio, después de los detalles del estudio, el diseño y productos bajo investigación se explicaron.

Consentimiento para publicación

No aplicable.

Disponibilidad de datos y material

Debido a la confidencialidad comercial de la investigación , los datos no incluidos en el manuscrito solo pueden estar disponibles a investigadores *bona fide* sujetos a un acuerdo de no-divulgación.

Fondos

Este estudio fue patrocinado por Elanco.

Intereses competentes

DK, KC, SC, MM, DC, SW, JD y SN son empleados de Elanco Animal Health.

Contribuciones de los autores

Todos los autores participaron en el diseño y finalización de los estudios y se involucraron en el manuscrito preliminar. Todos los autores leyeron y aprobaron el manuscrito final.

Detalles del autor

[1]Elanco Animal Health, Yarrandoo, NSW, Australia. [2]Elanco Animal Health, 2500 Innovation Way, Greenfield, IN 46140, EUA. [3]Elanco Animal Health, Schwarzwaldallee 215, CH-4058 Basilea, WRO-1032.2.58, Suiza. [4]Elanco Animal Health, Basingstoke, Hants, RU

Referencias

1. Hensel P, Santoro D, Favrot C, Hill P, Griffin C. Canine atopic dermatitis: detailed guidelines for diagnosis and allergen identification. BMC Vet Res. 2015;11:196.
2. Olivry T, DeBoer DJ, Favrot C, Jackson HA, Mueller RS, Nuttall T, et al. Treatment of canine atopic dermatitis: 2015 updated guidelines from the International Committee on Allergic Diseases of Animals (ICADA). BMC Vet Res. 2015;11:210.

3. Rolain JM, Franc M, Davoust B, Raoult D. Molecular detection of *Bartonella quintana*, *B. koehlerae*, *B. henselae*, *B. clarridgeiae*, *Rickettsia felis*, and *Wolbachia pipientis* in cat fleas, France. Emerg Infect Dis. 2003;9:338-42.
4. Rust MK, Dryden MW. The biology, ecology, and management of the cat flea. Annu Rev Entomol. 1997;42:451-73.
5. Dryden MW. Host association, on-host longevity and egg production of *Ctenocephalides felis felis*. Vet Parasitol. 1989;34:117–22.
6. Obendorf SK, Lemley AT, Hedge A, Kline AA, Tan K, Dokuchayeva T. Distribution of pesticide residues within homes in central New York State. Arch Environ Contam Toxicol. 2006;50:31-44.
7. Kuntz EA, Kammanadiminti S. Safety evaluation of lotilaner in dogs after oral administration as flavoured chewable tablets (Credelio™). Parasit Vectors (In press).
8. Cavalleri D, Murphy M, Seewald W, Drake J, Nanchen S. Assessment of the onset of lotilaner (Credelio™) speed of kill of fleas on dogs. Parasit Vectors (In press).
9. Murphy M, Cavalleri D, Seewald W, Drake J, Nanchen S. Laboratory evaluation of the speed of kill of lotilaner (Credelio™) against *Ixodes ricinus* ticks on dogs. Parasit Vectors (In press).
10. Cavalleri D, Murphy M, Seewald W, Drake J, Nanchen S. Assessment of the speed of flea kill of lotilaner (Credelio™) throughout the month following oral administration to dogs. Parasit Vectors (In press).
11. Cavalleri D, Murphy M, Gorbea RL, Seewald W, Drake J, Nanchen S. Laboratory evaluations of the immediate and sustained effectiveness of lotilaner (Credelio™) against three common species of ticks affecting dogs in Europe. Parasit Vectors (In press).
12. Marchiondo AA, Holdsworth PA, Fourie LJ, Rugg D, Hellmann K, Snyder DE, et al. World Association for the Advancement of Veterinary Parasitology (WAAVP) 2nd. ed.: Guidelines for evaluating the efficacy of parasiticides for the treatment, prevention and control of flea and tick infestations on dogs and cats. Vet Parasitol. 2013;194:84–97.
13. Blagburn BL, Dryden MW. Biology, treatment, and control of flea and tick infestations. Vet Clin Small Anim. 2009;39:1173-1200.
14. Robertson-Plouch C, Baker KA, Hozak RR, Zimmermann AG, Parks SC, Herr C, et al. Clinical field study of the safety and efficacy of spinosad chewable tablets for controlling fleas on dogs. Vet Ther. 2008;9:26-36.
15. Becskei C, De Bock F, Illambas J, Mahabir SP, Farkas R, Six RH. Efficacy and safety of a novel oral isoxazoline, sarolaner (Simparica™) in the treatment of naturally occurring flea and tick infestations in dogs presented as veterinary patients in Europe. Vet Parasitol. 2016;222:49-55.

16. Meadows C, Guerino F, Sun F. A randomized, blinded, controlled USA field study to assess the use of fluralaner tablets in controlling canine flea infestations. Parasit Vectors. 2014;7:375.
17. Cavalleri D, Murphy M, Seewald W, Drake J, Nanchen S. A randomized, controlled field study to assess the efficacy and safety of lotilaner tablets (Credelio™) in controlling fleas in client-owned dogs in European countries. Parasit Vectors (In press).
18. Cherni JA, Mahabir SP, Six RH. Efficacy and safety of sarolaner (Simparica™) against fleas on dogs presented as veterinary patients in the United States. Vet Parasitol. 2016;222:43-8.
19. Dryden MW, Ryan WG, Bell M, Rumschlag AJ, Young LM, Snyder DE. Assessment of owner-administered monthly treatments with oral spinosad or topical spot-on fipronil/(S)-methoprene in controlling fleas and associated pruritus in dogs. Vet Parasitol. 2013;191:340-6.
20. Halos L, Carithers DS, Solanki R, Stanford H, Gross SJ. Preference of dogs between two commercially available oral formulations of ectoparasiticide containing isoxazolines, afoxolaner or fluralaner. Open J Vet Medicine. 2015;5:25-9.
21. Plue RE, Jernigan AD, Acre KE, Coleman MW, Currin ST, Ellis AJ, et al. Field efficacy, safety and acceptability of ivermectin plus pyrantel in growing and adult dogs. In: Soll MD, editor. Proceedings of the Heartworm Symposium ’92. Batavia, IL: American Heartworm Society; 1992:205-8.

Leyenda de la figura

Fig 1 Porcentaje de perros primarios en cada grupo que estaban libres de pulgas en cada evaluación post-tratamiento en los Días 36, 60 y 90. *Diferencia entre grupos significativa, $P = 0.0323$

Tabla 1 Demografía de los perros enrolados y la distribución del número de perros en cada hogar. Los números se basaron en la población de seguridad de los perros del estudio, definidos como perros enrolados que recibieron al menos un tratamiento de estudio y regresaron al menos a una visita post-tratamiento

		**Lotilaner** (***n*** **= 197)**	**Afoxolaner** (***n*** **= 86)**
Edad (años)	Media ± DE	5.6 ± 4.0	5.5 ± 3.6
	Rango	0.2–16.0	0.3–15.0
Peso (kg)	Media ± DE	16.2 ± 11.4	18.8 ± 13.6
	Rango	2.0–46.1	2.2–65.0
Sexo	Hembra, intacta	25 (12.7%)	13 (15.1%)
	Hembra, esterilizada	70 (35.5%)	32 (37.2%)
	Macho, intacto	48 (24.4%)	15 (17.4%)
	Macho, esterilizado	54 (27.4%)	26 (30.2%)
Distribución del tamaño de los hogares (número de perros)			
	1	54 (46.6%)	25 (48.1%)
	2	43 (37.1%)	20 (38.5%)
	3	19 (16.4%)	7 (13.5%)

Abreviatura: DE, desviación estándar

Tabla 2 Datos del conteo de pulgas para cada grupo de tratamiento y el análisis estadístico de la reducción en el conteo de pulgas desde la línea base para cada grupo de tratamiento

		**Lotilaner**	**Afoxolaner**
Día 0	Media aritmética ± DE	70.7 ± 75.0	62.3 ± 61.8
	Rango[a]	10–251	10–251
	Media geométrica	42.3	41.8
Día 30	Media aritmética ± DE	0.7 ± 2.5	2.3 ± 5.5
	Rango	0–20	0–25
	Media geométrica	0.3	0.7
	% reducción	99.3	98.3
	Análisis estadístico	$t_{(105)} = 35.50$, $P < 0.001$	$t_{(47)} = 21.25$, $P < 0.001$
Día 60	Media aritmética ± DE	0.1 ± 0.4	0.3 ± 1.8
	Rango	0–3	0–12
	Media geométrica	0.0	0.1
	% reducción	99.9	99.8
	Análisis estadístico	$t_{(93)} = 38.19$, $P < 0.001$	$t_{(43)} = 28.10$, $P < 0.001$
Día 90	Media aritmética ± DE	0.0 ± 0.0	0.1 ± 0.6
	Rango	0–0	0–4
	Media geométrica	0.0	0.1
	% reducción	100	99.8
	Análisis estadístico	$t_{(173)} = 37.07$, $P < 0.001$	$t_{(43)} = 27.49$, $P < 0.001$

[a]Cualquier conteo >250 se le asignó el valor de 251

Abreviatura: DE, desviación estándar

Tabla 3 Análisis estadístico comparando los grupos de tratamiento con respecto a la media geométrica de los conteos de pulgas y la proporción de perros con cero pulgas

	**Conteo de pulgas**		**Perros con cero pulgas**	
Día 30	$t_{(143)}$ = 2.63	*P* = 0.0095	Tabla prob. = 0.0396	*P* = 0.1001
Día 60	$t_{(127)}$ = 1.44	*P* = 0.1530	Tabla prob. = 0.2493	*P* = 0.6795
Día 90	$t_{(125)}$ = 2.37	*P* = 0.0193	Tabla prob. = 0.0323	*P* = 0.0323
