# Supplementary material for: A randomized, controlled field study to assess the efficacy and safety of lotilaner flavored chewable tablets (Credelio™) in eliminating fleas in client-owned dogs in the USA
Source: Parasit Vectors. 2017 Nov 1;10:528. doi: 10.1186/s13071-017-2469-x (PMC5664423; doi:10.1186/s13071-017-2469-x)
Supplement: Supplementary file 2 — French translation of the Abstract. (PDF 43 KB) [file 13071_2017_2469_MOESM2_ESM.pdf]

Étude de terrain contrôlée, randomisée visant à évaluer l'efficacité et la sécurité d'un traitement par des comprimés à croquer aromatisés à base de lotilaner (Credelio™) contre les puces chez des chiens de compagnie aux États-Unis

Daniela Karadzovska[1], Kimberly Chappell[2], Shane Coble[2], Martin Murphy[3], Daniela Cavalleri[3], Scott Wiseman[4], Jason Drake[2]* et Steve Nanchen[3]

[1]Elanco Animal Health, Yarrandoo, NSW, Australie
[2]Elanco Santé animale, 2500 Innovation Way, Greenfield, IN 46140, États-Unis
[3]Elanco Santé animale, Schwarzwaldallee 215, CH-4058 Bâle, WRO-1032.2.58, Suisse
[4]Elanco Animal Health, Basingstoke, Hants, Royaume-Uni

*Correspondance : drake_jon_j@elanco.com

Adresse électronique :
Daniela Karadzovska : karadzovska_daniela@elanco.com
Kimberly Chappell : chappell_kim@elanco.com
Shane Coble : shane.coble@elanco.com
Martin Murphy : murphy_martin_gerard@elanco.com
Daniela Cavalleri : cavalleri_daniela_a@elanco.com
Scott Wiseman : wiseman_scott@elanco.com
Jason Drake : drake_jon_j@elanco.com
Steve Nanchen : nanchen_steve@elanco.com

Résumé

Contexte : des études précliniques ont montré que le lotilaner (Credelio™, Elanco), un nouvel agent de la classe des isoxazolines exerce un effet « knockdown » rapide sur les tiques et les puces après administration par voie orale chez le chien. De plus, cette efficacité persiste pendant au moins un mois après le traitement, avec une importante marge de sécurité. Une étude a été conduite sur le terrain afin de valider ces résultats précliniques.

Méthodes : les chiens ont été recrutés dans 10 cliniques vétérinaires aux États-Unis. Les foyers sélectionnés, comptant jusqu'à trois chiens dont un principal hébergeant au mois 10 puces ont été randomisés selon un rapport 2/1 pour recevoir le lotilaner (Credelio™, Elanco) à la dose minimale recommandée de 20 mg/kg, ou l'afoxolaner (Nexgard®, Merial), administré conformément à la notice, à la dose minimale de 2,5 mg/kg. Les traitements ont été délivrés aux propriétaires à J0, J30 et J60 pour administration par les propriétaires. Tous les chiens du foyer ont reçu le même traitement que celui du chien principal. Après l'inclusion, les puces et tiques ont été dénombrées chez les chiens principaux à J30, J60 et J90. L'appétence et la sécurité d'emploi des comprimés ont été évaluées pour tous les chiens.
Résultats : l'efficacité et l'innocuité ont respectivement été évaluées à partir de données de 111 et 197 chiens traités par lotilaner et de 50 et 86 chiens traités par l'afoxolaner. La diminution en pourcentage de la moyenne géométrique du nombre de puces dans le groupe lotilaner a respectivement été de 99,3 %, 99,9 % et 100 % à J30, J60 et J90 par rapport aux valeurs initiales, et de 98,3 %, 99,8 % et 99,8 % pour l'afoxolaner ($p < 0,001$ pour les deux groupes et tous les jours). A J90, aucune puce n'a été isolée chez 100 % des chiens traités par lotilaner et 93 % de ceux sous afoxolaner. Les tiques étaient trop peu nombreuses pour permettre une évaluation. Aucune différence n'a été observée en termes d'appétence entre les produits ($p = 0,2132$), avec un taux d'acceptation de respectivement 94 % et 96 % pour les traitements par lotilaner et afoxolaner distribués à la main, dans une gamelle vide ou avec un aliment. Les deux traitements ont été bien tolérés et ont soulagé les signes cliniques de dermatite par allergie aux piqûres de puces (DAPP) chez les chiens présentant des signes de DAPP lors de leur inclusion dans l'étude clinique.
Conclusion : une seule administration de lotilaner par le propriétaire a permis de réduire le nombre moyen de puces avec une efficacité supérieure à 99 % dans les 30 jours. L'administration de trois traitements mensuels consécutifs a permis de réduire de 100 % les infestations par les puces tout en réduisant de façon importante les signes de DAPP. Les comprimés aromatisés à base de lotilaner ont été facilement acceptés dans des conditions réelles de traitement. L'absence d'événements indésirables liés au traitement confirme l'innocuité du lotilaner chez le chien.
